# HTLV-1 Infection and Adult T-Cell Leukemia/Lymphoma—A Tale of Two Proteins: Tax and HBZ

**DOI:** 10.3390/v8060161

**Published:** 2016-06-16

**Authors:** Chou-Zen Giam, Oliver John Semmes

**Affiliations:** 1 Department of Microbiology and Immunology, Uniformed Services University of the Health Sciences, 4301 Jones Bridge Rd., Bethesda, MD 20814, USA; 2 Department of Microbiology and Molecular Cell Biology, The Leroy T. Canoles Jr Cancer Research Center, Eastern Virginia Medical School, Norfolk, VA 23501, USA; semmesoj@evms.edu

**Keywords:** HTLV-1, senescence, latency and persistence, RNF8, UBC13, K63-linked polyubiquitin, DNA damage response, genomic instability, adult T-cell leukemia

## Abstract

HTLV-1 (Human T-cell lymphotropic virus type 1) is a complex human delta retrovirus that currently infects 10–20 million people worldwide. While HTLV-1 infection is generally asymptomatic, 3%–5% of infected individuals develop a highly malignant and intractable T-cell neoplasm known as adult T-cell leukemia/lymphoma (ATL) decades after infection. How HTLV-1 infection progresses to ATL is not well understood. Two viral regulatory proteins, Tax and HTLV-1 basic zipper protein (HBZ), encoded by the sense and antisense viral transcripts, respectively, are thought to play indispensable roles in the oncogenic process of ATL. This review focuses on the roles of Tax and HBZ in viral replication, persistence, and oncogenesis. Special emphasis is directed towards recent literature on the mechanisms of action of these two proteins and the roles of Tax and HBZ in influencing the outcomes of HTLV-1 infection including senescence induction, viral latency and persistence, genome instability, cell proliferation, and ATL development. Attempts are made to integrate results from cell-based studies of HTLV-1 infection and studies of HTLV-1 proviral integration site preference, clonality, and clonal expansion based on high throughput DNA sequencing. Recent data showing that Tax hijacks key mediators of DNA double-strand break repair signaling—the ubiquitin E3 ligase, ring finger protein 8 (RNF8) and the ubiquitin E2 conjugating enzyme (UBC13)—to activate the canonical nuclear factor kappa-light-chain-enhancer of activated B-cells (NF-κB) and other signaling pathways will be discussed. A perspective on how the Tax-RNF8 signaling axis might impact genomic instability and how Tax may collaborate with HBZ to drive oncogenesis is provided.

## 1. HTLV-1 Infection and Adult T-Cell Leukemia

Human T-cell lymphotropic virus type 1 (HTLV-1) is a complex human delta retrovirus that infects 10–20 million people worldwide [1]. HTLV-1 Infection is mostly asymptomatic. However, via mechanisms that are not fully understood, 3%–5% of infected individuals develop a highly aggressive malignancy known as adult T-cell leukemia/lymphoma (ATL) decades after infection [2,3]. HTLV-1 infection also leads to several inflammatory and immune-mediated disorders, most notably HTLV-1-associated myelopathy (HAM)/tropical spastic paraparesis (TSP). ATLs are clinically classified as acute, lymphomatous, chronic, and smoldering types, with smoldering ATL possibly representing the early stage of the disease that often progresses to acute ATL over time [4]. There is also a pre-ATL state characterized by the presence of peripheral lymphocytes that display abnormal morphology resembling ATL cells.

The leukemic cells of ATL are monoclonal and harbor HTLV-1 proviral DNA at random chromosomal integration sites. Two viral regulatory proteins, Tax and HTLV-1 basic zipper protein (HBZ), encoded by the sense and antisense viral transcripts, respectively, are thought to play indispensable roles in the oncogenic process of ATL [3,5]. Tax is a potent activator of viral transcription and I kappa B kinase (IKK)/ nuclear factor kappa-light-chain-enhancer of activated B-cells (NF-κB) signaling. It also exerts pleiotropic effects on cell signaling. HBZ, by contrast, antagonizes many of the activities of Tax. A recent whole-genome/exome/transcriptome analysis of a large cohort of ATL patients has revealed recurrent activating mutations in phospholipase Cγ1 (PLCG1) (36%), protein kinase Cβ (PKCB) (33%), caspase recruitment domain-containing protein 11 (CARD11) (24%), disks large homolog 1 (DLG1) (24%), vav guanine nucleotide exchange factor 1 (VAV1) (18%), and cluster of differentiation 28 (CD28) (18%) in ATL samples [6]. These genetic alterations converge on the T-cell receptor-NF-κB and the CD28 co-stimulatory signaling pathways, and show remarkable functional overlap with the Tax interactome [6]. Tax expression, however, is frequently lost from ATLs (47/48 cases examined). By contrast, the expression of the HBZ antisense transcript is ubiquitous [6]. The loss of Tax expression from ATL cells suggests that the oncogenic effects of Tax are exerted early during ATL development, with HBZ playing a role in ATL maintenance [7,8,9,10]. Unlike other hematological malignancies, ATLs are noted for extensive genomic instability, a feature that has been fully borne out by the whole-genome analysis mentioned above, showing 59.5 structural variations/ATL sample and 7.9 point mutations/10^6^ bases, almost thrice that of multiple myeloma (21 chromosomal rearrangements/sample and 2.9 point mutations/10^6^ bases) [11]. Although the molecular basis for the genomic instability of ATL is not fully understood, earlier studies have implicated a link to Tax, which represses DNA damage repair (DDR) and disrupts mitotic processes [12,13,14,15].

## 2. HTLV-1 Viral Gene Expression and Regulation

In addition to structural proteins Gag, Pol, and Env, HTLV-1 encodes six viral accessory proteins, Tax, Rex, p12^I^, p13^II^, p30^II^, and HBZ from partially overlapping open reading frames (ORFs) in both directions of the viral genome. For a more recent review on p12^I^, p13^II^, and p30^II^, the reader is referred to this article [16]. P12^I^, a membrane-associated protein, appears to play a role in enhancing T-cell activation and signaling, although contradictory findings have also been described. A recent paper has reported p12^I^-mediated down-regulation of intercellular adhesion molecules ICAM-1 and ICAM-2, which is thought to mitigate autologous natural killer cell cytotoxicity for the infected CD4+ T-cells [17]. p12^I^ and its proteolytic cleavage product p8 are critical for HTLV-1 viral persistence and spread *in vivo*, and in keeping with these functions, p12^I^ has been reported to bind major histocompatibility complex (MHC) class I heavy chain and targets it for degradation [18]. The p13^II^ is an inner mitochondria membrane protein with anti-proliferation activity [19]. It also becomes ubiquitinated in the presence of Tax and translocates to the nucleus, where it disrupts Tax-CREB binding protein (CBP)/p300 interaction and inhibits viral and cellular transcription [20]. Its role in the HTLV-1 infection and replication cycle is not fully defined. The anti-proliferation activity of p13^II^ appears to be related to an interaction with farnesyl pyrophosphate synthetase [21] and the increased sensitivity to Ca^2+^-mediated stimulation and enhanced C2-ceramide-induced apoptosis of p13^II^-expressing T lymphocytes [22]. p30^II^ is a nuclear protein that functions as a post-transcriptional modulator of viral replication. Data suggest that p30^II^ retains the doubly-spliced tax/rex mRNA in the nucleus and thereby down-modulates viral gene expression by reducing the levels of Tax and Rex [16,23]. Rex regulates the transport of unspliced and singly-spliced mRNA, while Tax, the viral transcriptional activator, is thought to be the source of HTLV-1’s oncogenic potential. Tax expression is sufficient to effect cellular transformation in rodent fibroblasts and in primary human lymphocytes, and expression of Tax through a variety of promoters induces neoplasia in transgenic mice. In promoting viral replication, HTLV-1 Tax interacts with cellular regulators of homeostasis; these interactions ultimately perturb a number of basic cellular processes, many or all of which contribute to leukemia development. The major anti-sense HBZ mRNA, spliced HBZ (sHBZ), spans the open sequence between the *env* and the *tax/rex* ORFs. The region of the transcript complementary to the tax/rex mRNA is removed by splicing and, therefore, not expected to affect tax/rex mRNA by RNA interference. Similarly, a minor unspliced HBZ (usHBZ) transcript has its transcriptional start site upstream of the tax/rex region, and hence does not affect Tax/Rex. Both sHBZ and usHBZ mRNAs encode, respectively, basic domain-leucine zipper proteins with minor differences in their respective NH_2_-termini, and both forms of HBZ have been shown to negatively regulate Tax trans-activation [24] (see below). Importantly, the spliced HBZ protein and RNA are expressed in all ATL cells and can stimulate cell proliferation [5]. 

## 3. HTLV-1 Infection and Its Outcomes

### 3.1. HTLV-1 Transmission Requires Cell-to-Cell Contacts

HTLV-1 infection is highly dependent on cell propagation. Human transmission of HTLV-1 requires the transfer of virus-infected cells via breast-feeding, sexual intercourse, transfusion of cell-containing blood components, and needle sharing; all suggest a mechanism that depends upon cell-cell transfer. *In vitro*, HTLV-1 infects a wide variety of cells, including T and B lymphocytes, monocytes, endothelial cells, and fibroblasts. This is due in part to its use of a ubiquitous cell surface molecule, the glucose transporter 1, as the receptor for virus entry [25]. Other molecules, such as neuropilin-1 and heparan sulfate proteoglycans, also contribute to viral infection [26,27]. The broad tropism of HTLV-1 notwithstanding, its transmission requires cell-to-cell contact [28,29,30]. Cell-free HTLV-1 viral particles are poorly or not infectious directly [6]. The difference in efficiency between cell-mediated and cell-free infection is in the order of 10^5^ to 1 [31]. Cell-to-cell transmission of HTLV-1 occurs through the “virological synapse” formed in part through lymphocyte function-associated antigen 1 (LFA1) and ICAM1 [29,32]. Tax expression and ICAM1 engagement cause the microtutule polarization associated with the virological synapse. Tax is also localized to the region of the cell–cell contact formed between an HTLV-1 donor cell and its target cell, and at the vicinity of the microtubule-organizing center associated with the *cis*-Golgi [32,33]. Interestingly, it has been shown recently that dendritic cells exposed to free HTLV-1 particles not only become productively infected themselves (*cis*-infection), but can also rapidly transmit the virus to CD4+ T-cells (*trans*-infection) [34,35,36] (reviewed in [37]). HTLV-1 viral particles have also been found to be stored as carbohydrate-rich, biofilm-like extracellular assemblies that rapidly attach to target cells for virus transmission [38]. Finally, the presence of viral proteins in exosomes suggests a role for cell-derived microvesicles in HTLV-1 infection [39]. One might envision microvesicles as assisting virus transfer to naive cells, facilitating the delivery of bioactive viral proteins, or sequestration of immune-reactive viral proteins.

### 3.2. Evolution of HTLV-1-Infected T-Cells *In Vivo*

The details of the early events of acute HTLV-1 infection in humans are unknown. Analyses of chronic HTLV-1 infection in affected individuals have indicated that the HTLV-1 proviral DNA sequences show little nucleotide variations, suggesting that viral replication via error-prone reverse transcription occurs infrequently [40]. In asymptomatic carriers (ACs), HTLV-1 viral particles, mRNAs, and proteins, are virtually undetectable. The “latent” HTLV-1 genome is maintained in infected T-cells through limited mitotic division, presumably accompanied by occasional re-activation and *de novo* infection. ATL is usually characterized by the monoclonal expansion of a single leukemic cell that harbors the HTLV-1 proviral DNA integrated at a clone-specific chromosomal locus. Tax expression is largely silenced in ATL cells. This has been attributed to the negative selection of Tax-expressing cells by Tax-specific cytotoxic T lymphocyte-mediated killing [41,42,43].

### 3.3. Clonal Expansion of HTLV-1-Infected T-Cells *In Vivo*

Because very little HTLV-1 replication and gene expression can be detected in infected individuals, proviral DNA load (PVL) has been used as a quantitative measure of viral infection. Longitudinal studies of newly infected seroconverters and ACs have indicated that the clonality of HTLV-1-infected T-cells is more heterogeneous and unstable in the former [44]. Using the inverse-long PCR procedure to analyze PVLs and clonality of ATL and pre-diagnostic peripheral blood mononuclear cells (PBMCs) (3–8 years prior to ATL onset), Okayama *et al.* have reported that prior to the disease onset, there is a significant rise in PVLs. In one ATL case for which both leukemic and pre-diagnostic samples are available, pre-leukemic cells harboring the same integrated provirus as the leukemic cells could be detected 2, 5, and 8 years prior to ATL diagnosis, supporting the notion that persistent clonal expansion, selection, and evolution drive ATL development [45]. In a separate study, Umeki *et al.* have analyzed longitudinal samples collected over a period of more than a decade from a group of three Jamaican carrier children who acquired HTLV-1 perinatally [46]. The study indicates that the HTLV-1 PVLs are variable (10^2^–10^3^ copies/10^5^ PBMCs) in ACs. Some of these clones persisted for years, and two unique clones in one subject underwent significant expansion a decade or longer after the initial infection, causing PVLs to increase more than 40-fold, from 3 × 10^3^ to 1.3 × 10^4^ copies/10^5^ PBMCs. While the clonal expansion did not result in HAM/TSP or ATL, lymphadenopathy, seborrheic dermatitis, and hyperreflexia were observed in the subject [46].

More recently, high-throughput DNA sequencing has been used to characterize the chromosomal integration sites of HTLV-1 proviral DNA and the clonality of infected cells in ACs, and HAM/TSP and ATL patients (reviewed in [47]). These studies have demonstrated that the size of each proviral clone in ACs varies within the range of <1–10^3^ per 10^5^ PBMCs, and a large majority of infected cells harbor a single integrated provirus [48]. In agreement with this finding, in 91% of ATL cases, a predominant and presumably malignant T-cell clone containing one single provirus is detected [49]. An earlier study has shown that the integration pattern of an HTLV-1 vector devoid of viral genes in HeLa cells is randomly dispersed and shows a moderate preference for transcriptional start sites and CpG islands [50]. Similar integration site preferences were found *in vivo* (in infected individuals) and *in vitro* (cell culture-based infection) [51]. 

HTLV-1 clones that persist in chronically infected persons show little detectable Tax mRNA or protein expression *in vivo* and *ex vivo*, and silencing of Tax expression via 5’-long terminal repeat (LTR) methylation, 5’-LTR deletion, or nonsense mutations within its coding sequence is correlated with the expansion of malignant T-cells [52,53]. The 3’ region of the viral genome and the 3’-LTR, however, remain intact and unmethylated, thus favoring HBZ expression [53]. *In vitro* culture of infected but Tax-negative T-cells from donors induce Tax expression, but clones that remain Tax-negative in culture are more abundant [47]. Although Tax silencing correlates with clone abundance *in vivo*, the HTLV-1 proviral DNAs often integrate within genes and in transcriptionally active regions of the genome, and the integration properties of ATL clones are similar to those of ACs. Such an integration pattern is thought to favor HBZ expression. It also suggests that, upon integration, the chromosomal integration loci initially favor Tax and viral gene expression/replication, but are later silenced epigenetically when the infection enters into the chronic phase. While the loss of Tax and sense mRNA expression has been attributed to the negative selection pressure exerted by the host immune system (Tax-specific cytotoxic T lymphocytes (CTLs)) on virus-infected cells [41,42,43], the cellular senescence response triggered by Tax during productive infection in cell culture most likely also plays a role (discussed below) [54,55,56,57]. 

### 3.4. HTLV-1 Infection in Cell Culture 

HTLV-1 infection in cell culture is usually achieved by co-cultivation of naïve PBMCs with mitotically inactivated HTLV-1-producing cells [58,59] or by cell-free infection using vesicular stomatitis virus (VSV) G-pseudotyped viral particles [30]. As the efficiency of infection is poor and the detection of newly infected cells is technically challenging, studies of the outcome of HTLV-1 infection often involve long-term culture of infected PBMCs, with immortalization/transformation of virus-infected primary CD4+ T-cells as the experimental end-point [58]. As the early events of viral infection are difficult to study, they have not been examined in depth, and the mitogenic effects of HTLV-1 infection and Tax are assumed largely based on results from T-cell immortalization and tumor development in Tax-transgenic models that occur long after viral infection and Tax transgene expression [60,61,62]. In the meantime, with only a few exceptions [63], constitutive expression of Tax in cultured cell lines is virtually impossible to achieve, suggesting that in order for Tax to drive cell immortalization/transformation and oncogenesis, specific cellular alterations are needed or alternative viral factor(s) is (are) involved. 

### 3.5. HTLV-1 Infection in Rabbits 

Of the animal models of HTLV-1 infection, the rabbit model is perhaps the most amenable to experimentation, although none of the human diseases associated with HTLV-1 infection have been recapitulated therein [64,65]. With this system, Li *et al.* have shown that the tax/rex mRNA levels in PBMCs peak one week after inoculation of rabbits with γ-irradiated HTLV-1-produing cells [66]. They then rapidly declined to low levels when the infection progressed beyond the second week. As might be expected, gag-pol mRNA expression is coincident with, but at levels that are approximately one quarter that of the levels of tax/rex mRNA, and became mostly undetectable two to three weeks after infection. By contrast, HBZ mRNA levels were low at the start of the infection, but slowly increased and stabilized at four weeks and beyond. This is then followed by a rise in the proviral DNA load that mostly peaked at six weeks after inoculation. These results suggest that immediately after HTLV-1 infection, there is a strong selective pressure to silence viral gene expression, and only cells expressing Tax/Rex and HBZ at low and steady levels, respectively, persist. Whether the rapid decline in Tax/Rex and Gag-Pol expression soon after infection is due solely to the elimination of infected cells by CTL is unclear. As detailed below, the senescence response triggered by Tax may also play a key role in selecting for infected cells that express viral genes minimally.

### 3.6. Tax, NF-κB Hyperactivation, and Senescence 

As HTLV-1 causes ATL *in vivo* and transforms T-cells in cell culture, it is widely accepted that HTLV-1 infection causes T-cells to proliferate [3]. Because HTLV-1 Tax potently activates viral transcription, and NF-κB and other signaling pathways, it has been proposed that, via these activities of Tax, especially NF-κB activation, HTLV-1-infected T-cells are stimulated to proliferate, which eventually results in ATL. However, early studies describing the ability of Tax to compliment Ras but repress Myc in promoting anchorage-dependent growth clearly demonstrated that the cellular genetic background greatly influences the outcome of Tax expression [67,68]. 

We have used several lymphoid (Jurkat and SupT1) and non-lymphoid (HeLa and HOS) reporter cell lines to investigate the early cellular response to HTLV-1 infection in culture [54,55,69]. Contrary to the prevailing thinking that HTLV-1 infection leads to cell proliferation, our results show that most HTLV-1-infected lymphoid or non-lymphoid cells cease proliferation one cell division cycle after infection [54,55,69]. The infected cells express high levels of cyclin-dependent kinase inhibitors: p21^CIP1/WAF1^ (p21) and p27^KIP1^ (p27), develop mitotic abnormalities often with cytokinesis failure [56], and become arrested in senescence driven by Tax-mediated hyperactivation of NF-κB [57]. These results are in agreement with the induction of G1 cell cycle arrest seen with CD34+ hematopoietic progenitor cells infected by HTLV-1 in culture [70]. The dramatic rise in p21 and p27 in response to Tax is due to transcriptional up-regulation in conjunction with mRNA stabilization (of p21) [56,57,71], and protein stabilization (of p27) as a result of S-phase kinase-associated protein 2 (Skp2) degradation caused by the prematurely activated anaphase-promoting complex [56,72]. When NF-κB is repressed in HeLa or HOS cells by the stable expression of a degradation-resistant mutant of inhibitor of kappa B (IκBα), ΔN-IκBα, the senescence response triggered by HTLV-1 infection is mitigated [57], the cell lines that are chronically infected by HTLV-1 and express all viral proteins can be readily established [55,69]. Indeed, chronically infected HTLV-1-producing NF-κB-repressed HOS cell lines could transmit the virus to Jurkat T-cells [69]. These results suggest that most HTLV-1 infections induce a senescence response mediated by hyperactivated NF-κB. This is contrary to the belief that Tax-dependent NF-κB activation immediately promotes HTLV-1-infected T-cells to undergo clonal expansion, leading to ATL development. Thus, in order for Tax to promote oncogenesis, the cellular senescence response would have to be inactivated first [73]. Tax is also known to cause apoptosis in an NF-κB-dependent manner in a variety of lymphoid and non-lymphoid cell lines and experimental settings [74,75,76,77,78,79,80]. The senescence and apoptosis responses induced by Tax are mechanistically linked to NF-κB activation, possibly with the choice between senescence and apoptosis dependent on the genetic makeup or biological characteristics of the infected cells. It is reasonable to think that both senescence and apoptosis constitute cellular responses to potentially oncogenic NF-κB hyperactivation that occurs during productive HTLV-1 infection. Thus, in addition to CTL-mediated killing, the innate cellular mechanism that guard against NF-κB hyperactivation also drives the elimination of cells that are productively infected by HTLV-1, thereby allowing only “latently” infected cells with minimal to no viral (sense) gene expression to persist. The expansion of these cells will have to depend mostly on HBZ, a low level of Tax, somatic mutations that are mitogenic, or a combination of these three.

### 3.7. Does Cellular Senescence Facilitate the Spread of HTLV-1 to Innate Immune Cells? 

**Figure 1:**
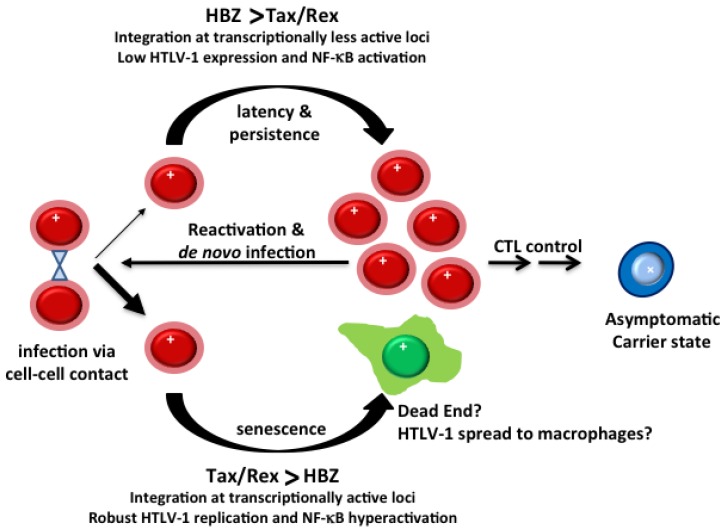
**Outcomes of HTLV-1 (Human T-cell lymphotropic virus type 1)** **infection.** Proviral integration sites determine the levels of Tax/Rex expression in infected cells. During productive infection, high levels of Tax/Rex override HTLV-1 basic zipper protein (HBZ)-dependent inhibition to promote productive viral replication, nuclear factor kappa-light-chain-enhancer of activated B-cells (NF-κB) hyperactivation, and cellular senescence. Approximately 98% of HeLa cells infected by HTLV-1 in culture become senescent [55]. When the levels of Tax/Rex are low, long terminal repeat (LTR) trans-activation, NF-κB activation, and senescence induction by Tax and viral mRNA nuclear export by Rex are inhibited by HBZ. As such, no viral structural proteins are expressed and the “latently” infected cells undergo mitotic expansion, likely propelled by HBZ and complemented by Tax that is expressed at subdued levels. The latent virus intermittently reactivates to initiate *de novo* HTLV-1 infection. The silencing of Tax and positive-sense viral mRNA expression are further selected by cytotoxic T lymphocyte (CTL) killing, resulting in an asymptomatic carrier state with little detectable serum levels of viral components.

As the majority of HTLV-1-infected cells become senescent, the question immediately arises as to why HTLV-1 retains such a self-limiting mechanism for viral replication. Is senescence just a cellular response to prevent NF-κB hyperactivation, and thus indirectly impacts HTLV-1 replication, or alternatively, could the biological properties of senescent cells be exploited by HTLV-1 for its unique lifestyle (Figure 1)? Studies of oncogene-induced senescence have demonstrated that factors secreted by senescent cells attract cells of the innate immune system, including natural killer (NK) cells, neutrophils, and macrophages, for their clearance (reviewed in [81]). As macrophages are highly proficient in spreading HTLV-1 to CD4+ T-cells, it would be interesting to investigate whether productively infected HTLV-1-producing cells that become senescent can efficiently recruit macrophages as conduits for transmitting HTLV-1. 

## 4. Oligoclonal Expansion of HTLV-1-Infected Cells

### 4.1. HBZ Mitigates the Senescence Response Induced by Tax and Promotes “Latent” HTLV-1 Infection 

HBZ antagonizes many of the activities of Tax, including LTR and NF-κB activation [57,82,83,84,85] (see below for additional details). We have found that, even though the majority of NF-κB-normal HeLa cells become senescent after HTLV-1 infection, a small population manages to continue to proliferate after infection and expresses low but detectable levels of Tax and Rex, albeit not Gag or Env [55]. In these cells, HTLV-1 LTR trans-activation by Tax continues at a reduced level, but NF-κB trans-activation is attenuated due to inhibition by HBZ. Interestingly, in these cells, gag-pol mRNA was found to localize primarily in the nuclei because Rex-mediated export of intron-containing mRNAs is blocked by HBZ [55]. Hence, during HTLV-1 infection, when Tax/Rex expression is robust and dominant over HBZ, productive infection ensues with expression of structural proteins and NF-κB hyperactivation, which induces senescence. When Tax/Rex expression is at a low level, HBZ attenuates Tax-driven canonical NF-κB activation [86], mitigates senescence induction, and allows infected cells that express viral genes minimally to proliferate and undergo clonal expansion [55,57]. In this “latent” state, HTLV-1-infected cells express Tax/Rex minimally or not at all, a low but steady level of HBZ, and no viral structural proteins. HBZ maintains viral latency by down-regulating Tax/Rex functions. Indeed, multiple lines of evidence support the notion that HBZ can promote “latently” infected cells to proliferate, and by so doing expand the reservoir of T-cells harboring the proviral genome [9,10]. It should also be noted that, when expressed at low levels, Tax may activate cell signaling to facilitate survival or expansion of infected cells. These findings are schematically summarized in Figure 1. Further investigation of the molecular aspects of HTLV-1 “latent” infection is needed to better understand the role in disease development.

### 4.2. Attenuation of NF-κB Activation and Loss of Senescence Response Facilitate Clonal Expansion of HTLV-1-Infected Cells *in Vitro*

Based on the premise that NF-κB hyperactivation by Tax drives senescence induction after viral infection, the inhibition of NF-κB activation and the loss of the p21/p27-driven senescence response should promote expansion of Tax-expressing HTLV-1-infected cells [87]. Indeed, HeLa and HOS cell lines expressing the degradation-resistant NF-κB super-repressor, ΔN-IκBα, can be productively infected [55]. Chronically infected NF-κB-repressed HOS clones in particular were able to transmit HTLV-1 to target T-cells with ease [69]. Thus, clonal expansion of HTLV-1-infected T-cells with significant Tax expression can occur after (a) attenuation/inhibition of IKK/NF-κB activation and/or (b) inactivation/loss of cellular senescence response. In this vein, it is interesting to note that the master transcriptional regulatory factor, forkhead box P3 (Foxp3), which is responsible for the development of T regulatory (Treg) cells [88], is a potent inhibitor of the transcriptional activities of cAMP response element-binding protein (CREB)/activating transcription factor (ATF), NF-κB, and nuclear factor of activated T-cells (NFAT) [89,90], and many HTLV-1-infected T-cells and ATL cells are CD4+, CD25^high^, and Foxp3+, and exhibit characteristics of Treg cells [91]. According to the model described above, Foxp3+ T cells would be better equipped to undergo clonal expansion after HTLV-1 infection. It is well established that p21 expression is highly induced in HTLV-1-transformed T-cell lines, but most likely is functionally inactivated or redirected to functions other than G1 cell cycle arrest [71,92], and is largely lost from ATL cells [93]. Further, in correlation with increased cell cycle entry, p27 protein expression is often greatly reduced in HTLV-1-transformed T-cell lines via the phosphatidylinositol-4,5-bisphosphate 3 (PI3)-kinase pathway [56,73]. ATL cell lines are therefore expected to be impaired in or to have lost the senescence response induced by HTLV-1 infection. Our unpublished results indicate that most ATL cell lines (ED, MT-1, ATL-55T, and TL-Om1) indeed continue to proliferate after re-infection by HTLV-1.

## 5. HTLV-1 Tax, Genomic Instability, and ATL Development

### 5.1. ATLs Are Associated with Extensive Genomic Instability 

Unlike other hematological malignancies, ATLs are characterized by extensive genomic instability, a feature that has been fully borne out by an integrated whole-genome analysis of ATL samples [6]. The molecular basis for the genomic instability of ATLs is linked to the HTLV-1 viral oncoprotein Tax, which, in addition to being a potent activator of viral transcription and IKK/NF-κB signaling, inhibits DNA double-strand break (DSB) repair, induces micronuclei formation, and disrupts spindle assembly and cytokinesis [3,12,94].

### 5.2. Tax and DNA Double-Strand Breaks

Micronuclei are extra-nuclear chromosomal fragments or whole chromosomes that form as a result of chromosome breaks (clastogenic events) or chromosome lagging during cell division. The induction of micronuclei formation by Tax has long been noted [13], and is associated with increased free DNA 3’ ends that are consistent with frequent occurrence of clastogenic DSBs [14]. In fact, genomic instability, as measured by the induction of micronuclei, represents one of the few differences in the biological activity of HTLV-1 Tax1 *versus* HTLV-2 Tax2 [15]. Tax expression can also increase the rate of gene amplification, as revealed by the *N*-(phosphonoacetyl)-L-aspartate-resistance (PALA) assay, which measures increased copy number of the carbamyl phosphate synthetase/aspartate transcarbamylase/dihydro-orotase (CAD) gene caused by DNA recombination following DSBs [95].

Mechanistically, there have been numerous Tax activities reported to result in genomic instability (summarized in Table 1). Early on, Tax has been found to repress the expression of DNA Polβ, a DNA polymerase involved in base excision repair [96]. Recent studies have found the expression of Tax to result in the accumulation of reactive oxygen species (ROS) [80] and NO [97], each of which induces DSBs. Tax has also been shown to directly repress the cellular DDR (for a review see [12]). We have described that Tax induces the formation of DNA damage-independent nuclear foci that contain DDR mediators such as breast cancer 1 (BRCA1), checkpoint kinase 2 (CHK2), DNA-dependent protein kinase (DNA-PK), and mediator of DNA damage checkpoint protein 1 (MDC1) [98,99,100,101]. The recruitment of one of these factors, MDC1, was competitive with the formation of DNA damage-induced foci and suggests that Tax represses cellular DDR via sequestration of MDC1 [98]. Whether Tax directly induces DSBs, inhibits the repair of DSBs, or both, has not been fully delineated. Our recent data showing that Tax hijacks DDR signal transducers, the ubiquitin E3 ligase, ring finger protein 8 (RNF8), and the ubiquitin E2 conjugating enzyme, UBC13, for IKK/NF-κB activation [102] (see below) may begin to provide a clear mechanistic understanding for how HTLV-1 infection leads to DNA damage, genomic instability, and ATL development.

### 5.3. Tax Hijacks Key Mediators of DNA Damage Repair to Activate TAK1, IKK/NF-κB, and Other Kinases 

**Figure 2:**
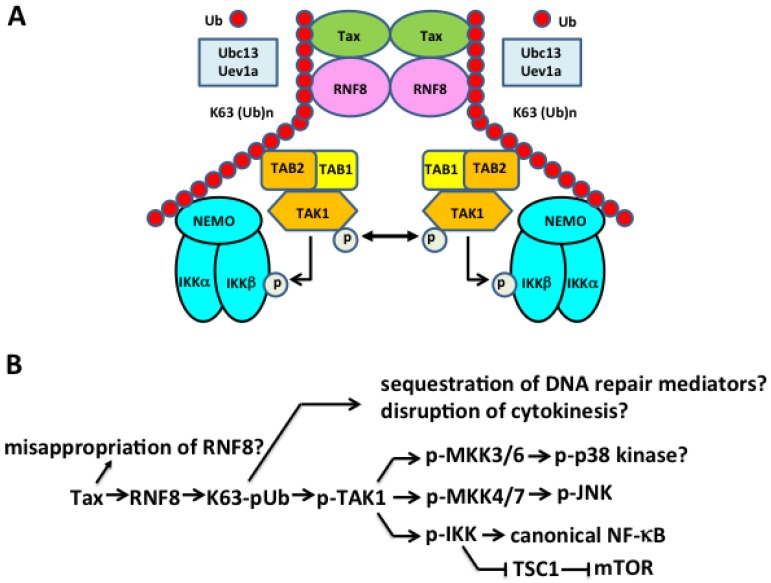
**Tax hijacks the ubiquitin E3 ligase, ring finger protein 8** (**RNF8) and E2 conjugating enzyme** **UBC13: Uev1A/Uev2 to activate transforming growth factor beta (TGFβ)-activated kinase** **1** (**TAK1), I kappa B kinase (IKK), canonical** **NF-κB** **and other signaling pathways.** (**A**) Tax directly interacts with and stimulates RNF8 and Ubc13:Uev1A/2 to assemble long lysine 63-linked polyubiquitin (K63-pUb) chains, which then serve as the signaling scaffolds for K63-pUb-binding TAK1 and IKK to convene and become activated. As NF-κB essential modulator (NEMO) interacts weakly with K63-pUb chains, the direct interaction between Tax and NEMO may mediate IKK recruitment to and activation by TAK1. (**B**) The Tax-RNF8 signaling axis drives increased assembly of K63-linked polyubiquitin chains, which activate TAK1. TAK1, in turn, signals the activation of MKKs and IKK, and downstream p38 kinase, c-Jun N-terminal kinase (JNK), mammalian target of rapamycin (mTOR), and the canonical NF-κB pathway. The misappropriation and aberrant activation of RNF8 and the increased assembly of K63-linked polyubiquitin chains may sequester key mediators of DNA damage repair and cytokinesis to induce genomic instability in the forms of DNA double-strand breaks (DSBs) and chromosome aneuploidy.

Polyubiquitin chain assembly is a post-translational modification by which protein-linked or unanchored polyubiquitin chains are assembled stepwise via three classes of enzymes, ubiquitin E1 activating enzymes, E2 conjugating enzymes, and E3 ligases. Lysine 48 (K48)-linked polyubiquitin targets proteins for proteasome-mediated degradation, while lysine 63 (K63)-linked and linear polyubiquitins are crucial in regulating a variety of cellular processes including cytokine-mediated IKK/NF-κB activation, DDR signaling, and cytokinesis [103,104,105]. Advances over the past decade have demonstrated that upon T- or B-cell receptor engagement, or stimulation by cytokines such as tumor necrosis factor alpha (TNFα) and interleukin-1 (IL-1), or pathogen-associated molecules such as bacterial lipopolysaccharide (LPS), members of the TNF receptor-associated factor (TRAF) family such as TRAF6, TRAF2/5, and cIAP1/2 become recruited to the receptors and interact with ubiquitin E2 conjugating enzymes, UBC13:Uev1a/Uev2, to assemble free or protein-anchored K63-linked polyubiquitin (K63-pUb) chains (reviewed in [106]). The IKK kinase, transforming growth factor beta (TGFβ)-activated kinase 1 (TAK1), and IKK both contain ubiquitin-binding subunits (TAK1-binding protein 2/3 (TAB2/3) and NF-κB essential modulator (NEMO), respectively) that facilitate their recruitment to K63-pUb chains where TAK1 undergoes auto-phosphorylation/activation [106]. Activated TAK1 then phosphorylates and activates IKK in its vicinity. IKK in turn phosphorylates IκBα, targeting it for K48-linked polyubiquitination and proteasomal degradation, thereby activating the canonical NF-κB pathway. Interestingly, Ed Hahaj’s lab has previously reported that conjugation of Tax by K63-pUb correlates with IKK activation, and requires the ubiquitin E2 conjugating enzyme UBC13 [107]. Indeed, mouse embryo fibroblasts containing bi-allelic deletion of the *ubc13* gene are deficient in supporting Tax-driven NF-κB activation [107]. Additional evidence implicating the importance of K63-pUb in Tax-dependent IKK/NF-κB activation came from *in vitro* assays showing that the K63R mutant of ubiquitin could inhibit IKK activation by Tax in cytosolic extract of Jurkat T-cells [108]. These results support the notion that K63-pUb assembly plays an important role in Tax-mediated IKK/NF-κB activation. Finally, using both cell-based assays and *in vitro* systems reconstituted from purified proteins, we have demonstrated that Tax hijacks and aberrantly activates the ubiquitin E3 ligase, RNF8, and its associated E2 conjugating enzymes, UBC13:Uev1a/Uev2—transducers of DSB repair signaling and cytokinesis regulation—to assemble K63-pUb chains for TAK1 and IKK/NF-κB activation [102]. These results indicate that IKK/NF-κB activation and DDR repression by Tax are likely connected via RNF8 (Figure 2A). It is conceivable that the hijacking of RNF8 by Tax and the ensuing over-production of misdirected K63-pUb chains result in a defect in DDR, thus driving the genomic instability in HTLV-1-infected T-cells. 

A most recent report by Wang *et al.* suggests that Tax itself is a ubiquitin E3 ligase that, together with a group of E2 enzymes including UBCH7, UBCH5b, UBCH5c, and UBCH2, assembles mixed-linkage polyubiquitin chains for IKK activation in a UBC13- and TAK1-independent manner [109]. This conclusion contrasts with published results from multiple laboratories showing the importance of TAK1 and UBC13 in Tax-driven NF-κB activation [102,107,108,110,111,112]. As mentioned above, Shibata *et al.* have previously shown that K63R and K0 mutants of ubiquitin block IKK activation by Tax in cell-free assays and concluded that the assembly of K63-pUb chains is crucial for Tax-mediated IKK activation [108]. This is the simplest interpretation of the data, and is in line with published literature [102,107,108,110,111,112] and the well-established mechanism by which IKK is activated. While Wang *et al.* confirmed the same observation, they interpreted the data in a more complex model, suggesting that K63R-Ub is a less efficient substrate for the assembly of mixed-linkage polyubiquitin chains by Tax. It should be pointed out that no RING domain or HECT domain commonly found in E3 ligases can be discerned in Tax. Whether Tax belongs to an entirely new class of E3 ligases that utilize multiple E2s to assemble mixed-linkage polyubiquitin chains, as claimed, remains to be confirmed.

Finally, RNF8, along with RNF168, are key mediators of the cellular response to DNA damage (for review see [113,114]). A hallmark feature of the cellular response to DSBs is the coordinated sequential formation of repair complexes into foci, the biogenesis and subsequent removal of which reflects repair efficiency. One of the early events in the formation of repair foci is ataxia telangiectasia mutated (ATM)-mediated phosphorylation of MDC1 and subsequent stabilization of repair complexes. RNF8 is recruited to sites of DSBs via interaction between its FHA domain and phosphorylated MDC1 [115]. The subsequent ubiquitination of local histones is required for assembly of p53-binding protein 1 (53BP1) and BRCA1 via K63-linked polyubiquitin chains and repair of DNA breaks [116]. RNF8 also ubiquitylates nibrin (Nbs1), a member of the Mre11/Rad50/Nbs1 (MRN) complex, to promote its binding to DSBs to facilitate repair via homologous recombination [117]. Underscoring the interplay between RNF8 and MDC1, subsequent degradation of MDC1 via sumoylation is a second critical determinant of homologous recombination [118]. Thus, it is likely that the competitive recruitment of MDC1 away from DNA damage foci is mediated through the sequestration or aberrant activation of RNF8 by Tax.

### 5.4. The Pleiotropic Effect of Tax on Cell Signaling Explained? 

Tax potently and specifically activates viral transcription by binding the basic domains of CREB/ATF-1 and contacting the G/C-rich sequences flanking the viral cAMP response elements in the Tax-responsive 21-bp repeat enhancer to form a ternary Tax:CREB/ATF-1:21-bp repeat nucleoprotein complex. In this ternary complex, Tax further recruits transcriptional co-activators/histone acetylases, CBP/p300 and p300/CBP-associated factor (P/CAF), and transducers of regulated CREB (TORCs) [119] to establish a nucleosome-free region for potent HTLV-1 viral mRNA transcription (reviewed in [120]). 

Tax is notorious for exerting pleiotropic influence on cell signaling. It activates both the canonical and non-canonical NF-κB pathways, the transcriptional activities of activator protein 1 (AP1), the serum response factor (SRF), and the NFAT, and the kinase activity of the mTOR, *etc.* [3]. Because of the many roles K63-pUb and TAK1 play in cell signaling, the hijacking and inappropriate activation of RNF8 by Tax and the resultant over-production of K63-pUb chains may provide a simple explanation for many of the biological effects of Tax (summarized in Figure 2B and Table 1). Indeed, the activation of JNK phosphorylation by Tax and RNF8 *in vitro* (via TAK1 and presumably the mitogen-activated protein kinase kinase MKK7) is consistent with earlier studies showing AP1 activation by Tax and JNK activation in HTLV-1-transformed cells [121]. Further, TAK1, IKKα, and IKKβ each control multiple signaling pathways that are NF-κB-unrelated [122]. TAK1 is known to activate MKK3/6 and MKK4/7, which activate downstream p38 kinase and JNK, respectively. IKKβ can phosphorylate and inactivate the tuberous sclerosis 1 (TSC1) tumor suppressor complex [123] that represses the serine/threonine kinase, the mTOR, leading to mTOR activation [123]. The implied activation of AP1, SRF, NFAT, and mTOR by Tax via the Tax⇒RNF8⇒K63-pUb chains⇒TAK1 signaling axis awaits further validation (Figure 2B). 

A recent study has shown that, during IL-1β signaling, linear polyubiquitin assembled by a unique E3 ligase complex, known as linear ubiquitin assembly complex (LUBAC), consisting of HOIL-1-interacting protein (HOIP), heme-oxidized IRP2 ubiquitin ligase-1 (HOIL-1), and Shank-associated RH domain interactor (Sharpin), becomes covalently attached to K63-pUb to form K63/M1-hybrid pUb [124]. Because NEMO has 100-fold higher binding affinity for M1-pUb than for K63-pUb, it is proposed that TAK1 and IKK, respectively, are recruited to K63-pUb and M1-pUb of the hybrid pUb such that signaling between these two kinases can occur. Tax is known to bind NEMO directly. It has been reported that the interaction between Tax and NEMO in cell-free extract is not affected by polyubiquitination [108]. Furthermore, the ubiquitin-binding defective mutants (D311N and L329P) of NEMO, while deficient in mediating NF-κB activation by TNFα, continue to support Tax-mediated NF-κB activation [110]. The direct interaction between Tax and NEMO may obviate the need for linear polyubiquitin in IKK recruitment to and activation by TAK1.

### 5.5. HTLV-1, Tax, and Chromosome Instability

Centrosomes, the microtubule organizing centers (MTOCs), are responsible for bipolar mitotic spindle formation and proper segregation of chromosomes during mitosis. Centrosome duplication occurs once in every cell division cycle (centrosome duplication “licensing”) and is tightly regulated. Centrosome amplification can lead to multipolar spindle formation and unequal chromosome segregation. Tax has been shown to cause centrosome amplification or fragmentation. This has been hypothesized as a contributing factor to the development of aneuploidy in ATL cells. The mechanism underlying Tax-mediated centrosomal abnormality is not entirely resolved. Interaction between Tax and Ran-specific binding protein 1 (RanBP1) that results in a disruption of Ran-RanBP1 regulation of centriole cohesion has been proposed [125]. A role of NF-κB in Tax-mediated centrosomal amplification has also been suggested [125]. Rootletin (TaxBP121), a protein with extensive coiled-coil structure previously identified in yeast 2-hybrid screen as a Tax-binding partner, has been shown to inhibit centrosome duplication [126]. TaxBP121 knockdown or putative inactivation through interaction with Tax, leads to centrosome hyperamplification. Another model for Tax-induced chromosome instability (CIN) posits that a direct interaction between Tax and human homolog of MAD1 (HsMAD1), a critical component of the spindle checkpoint, causes spindle assembly checkpoint defect [127], which allows mitosis to proceed even though proper attachment of sister chromatids to the mitotic spindle is impaired, thus causing uneven distribution of chromosomes and aneuploidy. However, Tax-expressing HTLV-1-transformed T-cells arrest in metaphase after treatment with the microtubule-disrupting agent, nocodazole, suggesting that the spindle checkpoint defect caused by Tax is subtle, if it happens at all [128,129]. RNF8 has an established non-enzymatic role in the unfolding of chromatin to facilitate DDR factor recruitment to DSBs [130]. In addition, RNF8 targets septins for ubiquitination and localizes to centrosomes [131]. Interestingly, RNF8-mediated polyubiquitination regulates exit from mitosis, and over-expression of RNF8 resulted in a delay of mitotic exit, unresolved cytokinesis, and multinucleated cells [132]. Again, it is tempting to speculate that the Tax–NF8 nexus is responsible for the reported effects of HTLV-1 on chromosomal integrity and multinucleation. Which of these mechanisms eventually can account for the cytokinesis defects and chromosome aneuploidy associated with Tax awaits further investigations. Finally, the pX region of HTLV-1 genome has been shown to contain a binding site for the CCCTC-binding factor (CTCF), a cellular protein involved in organizing high-order chromatin structure [133]. CTCF binding to the viral binding site sharply defines the border of epigenetic modifications in proviral DNA and acts as an enhancer blocker [134]. Interestingly, CTCF binding also regulates HTLV-1 mRNA splicing and mediates interaction between CTCF-binding sites in HTLV-1 proviral DNA and adjacent chromosomal regions [134]. How the long-range interaction between viral and cellular CTCF-binding sites and the ensuing chromatin looping contribute to the regulation of the HTLV-1 replicative cycle and the genomic instability in ATL cells remains to be explored. 

## 6. HBZ and ATL Development

### 6.1. HBZ Gene Expression

The *HBZ* gene is located at the 3’ end of the viral genome. HBZ RNA is synthesized from the minus or antisense strand of the viral genome, and is of the opposite polarity of the major HTLV-1 viral transcript. HBZ RNA exists in both spliced and unspliced forms. The sHBZ transcript is predominant in ATL cells, and it and its protein product are commonly referred to as HBZ. The protein product of the unspliced HBZ transcript is known as usHBZ. It is slightly larger than HBZ in size and contains a stretch of seven additional NH_2_-terminal amino acid residues, MVNFVSV. The usHBZ has a much shorter half-life compare to HBZ and is observed at lower concentrations in HTLV-1 infection. Tax has been found to up-regulate HBZ expression and this up-regulation is influenced by the HTLV-1 integration sites [135].

### 6.2. HBZ RNA, HBZ, and usHBZ

Both usHBZ and HBZ antagonize the activities of Tax at the levels of LTR *trans*-activation and NF-κB activation [86]. However, HBZ can stimulate T-cell proliferation, while usHBZ cannot [24]. HBZ expression is regulated by three specificity protein 1 (SP1) binding sites in a TATA-less promoter located in the 3’-LTR of the proviral DNA [24]. Interestingly, both HBZ and HBZ RNA have been shown to promote T-cell proliferation [10]. Thus, three separate molecules: HBZ, HBZ RNA, and usHBZ encoded by the antisense strand of the HTLV-1 genome function to regulate viral gene expression and the proliferation and persistence of provirus-harboring T-cells. The roles of usHBZ in viral replication, persistence, and pathogenesis remain unclear.

### 6.3. HBZ Antagonizes Many of the Activities of Tax

For details of the domain organization and activities of HBZ, readers are referred to several excellent reviews [5,136,137]. HBZ is a basic domain leucine zipper protein. Via its leucine zipper domain, HBZ interacts with and disrupts the DNA-binding or transcriptional activities of CREB-2, JunB, and c-Jun (AP1). It also binds the KIX domain of CBP/p300 and inhibits its HAT activity, thus blocking Tax-driven viral mRNA expression. HBZ, however, interacts with JunD and activates JunD-mediated transcription. As mentioned above, HBZ down-regulates the nuclear export of full-length and singly spliced HTLV-1 mRNAs by Rex to inhibit the production Gag, Gag-Pol, and Env proteins. HBZ also dampens NF-κB activity by preventing NF-κB binding to DNA and inducing p65/RelA degradation, thus mitigating the cellular senescence response triggered by Tax-driven NF-κB hyperactivation. These activities of HBZ promote the establishment of latent HTLV-1 infection and facilitate the persistence of infected cells (Figure 1). A comparison between Tax and HBZ activities and functional consequences is shown in Table 1.

### 6.4. HBZ Promotes T-Cell Proliferation

Soon after ATL cells were found to persistently express HBZ, but not Tax, both HBZ RNA and protein were shown to stimulate T-cell proliferation. Recent data indicate that HBZ protein additionally promotes apoptosis induction, while HBZ mRNA prevents it, in part via up-regulation of the *survivin* gene [138]. Importantly, CD4+ T lymphocyte-specific expression of the HBZ transgene in mice induces T-cell lymphoma and systemic inflammation [8]. The HBZ transgene also directly stimulates the expression of Foxp3 in mouse CD4+ T-cells, thus providing an explanation for why ATL cells are often Foxp3+ [139]. The expression of HBZ in CD4+ T-cells shows a positive correlation with the expression of oncomiRs, which have been associated with a wide range of oncogenic activities [140]. HBZ protein also assists JunD in the transcriptional activation of human telomerase reverse transcriptase (hTERT) [141]. Recent results indicate that HBZ protein can target the retinoblastoma protein (Rb)/ E2F transcription factor 1 (E2F1) complex to activate the transcription of genes under E2F1 control that are critical for DNA replication and cell cycle progression [138]. The activation of E2F-regulated genes by HBZ promotes both T-cell proliferation and apoptosis. These results clearly demonstrate the growth-promoting and oncogenic properties of HBZ, and explain why HBZ is persistently expressed in ATL cells. Finally, HBZ has been found to induce the expression of a co-inhibitory immune receptor molecule, T-cell receptor with immunoglobulin and ITIM domains (TIGIT), to facilitate IL-10 production, possibly to suppress antiviral immune responses [142].

## 7. Concluding Remarks and Future Perspectives

### 7.1. The Balance between Tax and HBZ Regulates Viral Latency and Persistence

**Figure 3:**
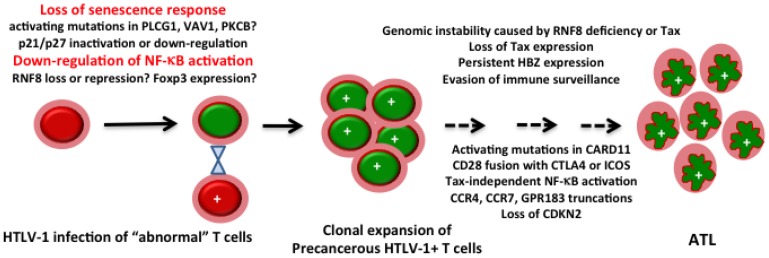
**A model for** **adult T-cell leukemia/lymphoma** **(ATL)** **development.** Loss of senescence response and/or inhibition or down-regulation of NF-κB activation (cells marked with green nuclei) drives clonal expansion of HTLV-1-infected cells (each marked with a “+” sign). Some of these cells, presumably precancerous, continue to evolve, propelled by the genomic instability caused by either Tax or the loss of RNF8, eventually giving rise to ATL. The frequent genetic alterations detected in ATLs, such as activating point mutations in phospholipase Cγ1 (PLCG1), vav guanine nucleotide exchange factor 1 (VAV1), and protein kinase Cβ (PKCB), activating point mutations and deletions in caspase recruitment domain-containing protein 11 (CARD11), CTLA4/CD152–CD28, and inducible T-cell COStimulator (ICOS)/CD278–CD28 fusions, homozygous deletion of cyclin-dependent kinase inhibitor 2A (CDKN2), and chemokine (C-C motif) receptor 4 (CCR4), CCR7, and G protein-coupled receptor 183 (GPR183) truncations, are incorporated into this model to stimulate discussions and suggest future experiments.

It has become abundantly clear over the past few years that HTLV-1 has evolved a replicative strategy that is unique among retroviruses. By encoding HBZ and HBZ RNA from promoters extrinsic of the viral LTRs (thus allowing expression of genes that are largely independent from viral replication and Tax *trans*-activation), HTLV-1 succeeds in (a) down-regulating Tax/Rex-mediated viral gene expression and replication; (b) mitigating the senescence response triggered by hyperactivated NF-κB; (c) down-regulating cellular immune responses [142]; and (d) promoting proliferation and survival of infected T-cells. These activities of HBZ and HBZ RNA facilitate evasion from immune detection and drive the persistence and expansion of “latently” HTLV-1-infected cells. The immortalization/transformation of T-cells by HTLV-1 that gives rise to T-cell lines such as MT2, MT4, and C8166 that express Tax at high levels occurs rarely, and most likely requires specific cellular changes that inactivate the senescence response induced by Tax (Figure 3).

### 7.2. The Indispensable Role of Tax and HBZ in ATL Development

With the ubiquitous expression of HBZ in ATL cells and the many roles of HBZ in viral persistence, growth stimulation, and tumor development firmly established, it would appear that Tax should relegate its title as the HTLV-1 viral oncogene. A critical review of the most current literature, however, indicates otherwise: Two of the most striking features of ATL revealed by the whole genome sequence analysis [6] are connected to Tax: (1) the extensive genomic instability of ATL; and (2) the functional overlap between the pathways and molecules that Tax is known to activate (Figure 2B) and the genetic alterations in ATLs, which converge on signaling molecules in the TCR-NF-κB and the CD-28 co-stimulatory pathways (Figure 3).

The hijacking and aberrant activation of RNF8 not only explains the pleiotropic effect of Tax on cell signaling (e.g., activation of TAK1, IKK/canonical NF-κB, and JNK), but likely can also account for the genomic instability induced by Tax (Figure 2B). Evidence in support of this hypothesis should be forthcoming. Based on current information, senescence induction by HTLV-1 can be lessened through the inactivation of the cellular mediators of the senescence response (p21 and p27) or the inhibition of NF-κB activation (Figure 3). One can easily imagine that during the course of HTLV-1 persistence, viral infection may accidentally take place in T-cells whose senescence mediators become inactivated, e.g., via activating mutations in PLCG1, PKCB, VAV1, or FYN that frequently recur in ATL [6] (Figure 3). Of note, VAV1 lies upstream of PLCG1 and PI3K and its activation causes the phosphorylation and inactivation of forkhead box protein O1 (FOXO1)—a transcription factor critical for p27 expression—to down-regulate p27 and promote T-cell proliferation [143]. It is conceivable that, with activating VAV1 mutations, HTLV-1 infection and significant Tax expression can occur without triggering an overt senescence response. The direct dysregulation of RNF8 by Tax can then promote genomic instability by causing a deficit in DSBs repair (Figure 2B). Since activating mutations in PLCG1 and PKCB also promote NF-κB activation, they may supplant many of the functions of Tax to facilitate the eventual silencing of Tax expression (Figure 3). When Tax expression becomes silenced during ATL development, the loss of senescence mediators could facilitate the development of Tax-independent NF-κB activation via CARD-11 mutations (Figure 3). In another scenario, clonal expansion of HTLV-1-infected T-cells may be facilitated by RNF8 down-regulation (which moderates TAK1 and NF-κB hyperactivation and is expected to dampen senescence induction). After Tax expression is silenced or lost, the down-regulation of RNF8 is expected to continue to cause genomic instability in cells that are propelled to proliferate by HBZ. In this vein, many of the genetic alterations such as chemokine (C-C motif) receptor 4 (CCR4), CCR7, and G protein-coupled receptor 183 (GPR183) truncations, intragenic deletions in CARD11, and gene rearrangements such as CTLA4/CD152–CD28 and and inducible T-cell COStimulator (ICOS)/CD278–CD28 fusions [6], may very well be the consequences of the genomic instability directly or indirectly induced by Tax. These scenarios are not mutually exclusive and may occur at different stages of HTLV-1 infection and disease progression. Finally, the Tax/RNF8-related genomic instability and the senescence-escaping activating mutations may prove to be the Achilles’ heel that can be exploited and targeted to develop effective treatment approaches for ATL.

## Figures and Tables

**Table 1:** A comparison of the activities and functional consequences of Tax and HTLV-1 basic zipper protein (HBZ).

Tax Activities	Functional Consequences	HBZ Activities	Functional Consequences
CREB, CBP/p300, P/CAF, TORC interaction	Activate viral transcription	CREB, CBP/p300 interaction	Suppress viral gene expression
Association with MTOC	Promote formation of virological synapse and cell–cell transmission	Rex inhibition	Suppress viral gene expression and particle production
RNF8, UBC13 interaction and activation	Stimulate K63-linked polyubiquitin chain assembly	NF-κB DNA-binding disruption and p65/RelA degradation	Suppress Tax-mediated canonical NF-κB activation
TAK1, IKK, MKK, JNK, mTOR, *etc.* activation	Activate c-Jun/AP and SRF		Prevent senescence induction
Canonical NF-κB activation	Induce expression of cytokines, cytokine receptors, adhesion molecules, anti-apoptotic factors, *etc.*		Promote viral latency and persistence of virus-infected cells
p21^WAF1^ and p27^KIP1^ up-regulation	Induce senescence		
NIK, p100 interaction Non-canonical NF-κB activation	Induce expression of cytokines, cytokine receptors, adhesion molecules, anti-apoptotic factors, *etc.*	E2F1 activation	Promote cell proliferation and apoptosis
		Survivin up-regulation (HBZ RNA)	Prevent apoptosis
CDK 2/4 activation E2F1 activation	Promote cell cycle progression	Onco-miRs activation	Promote cell proliferation
Cyclin D1 activation P53, Rb, DLG1 inactivation		hTERT activation BDNF/TrkB activation	
PCNA activation		Wnt5a, JunD activation	
		CENP-B repression	
hTERT activation	Promote cell immortalization	Foxp3 induction and functional inactivation	Modify T-cells
P53 inactivation Sequestration of DDR mediator MDC1 Inactivation of DDR mediators CHK1 and CHK2 Suppression of DNA Polβ expression	Induce genomic instability	Bim repression via Foxo3a IFNγ repression Activation of mTOR/suppression of autophagy	Promote cell survival during stress response
Activation of APC/C	Promote aneurploidy, cytokinesis defect, and senescence	TIGIT induction	Impair antiviral immunity and promote immune evasion
RANBP1, TaxBP2 (Rootletin isoform 2) interaction	Promote centrosome amplification or fragmentation		

CREB: cAMP response element-binding protein; CBP/p300: Tax-CREB binding protein (CBP)/p300; P/CAF: p300/CBP-associated factor; TORC: transducers of regulated CREB; MTOC: microtubule organizing centers; RNF8: ring finger protein 8; UBC13: ubiquitin E2 conjugating enzyme; NF-κB: nuclear factor kappa-light-chain-enhancer of activated B-cells; TAK1: TGFβ)-activated kinase 1; IKK: I kappa B kinase; MKK: mitogen-activated protein kinase kinase; JNK: c-Jun N-terminal kinase; mTOR: mammalian target of rapamycin; AP: activator protein; SRF: serum response factor; NIK: NF-κB-inducing kinase; E2F1: E2F transcription factor 1; CDK: cyclin-dependent kinase; Rb: retinoblastoma protein; DLG1: disks large homolog 1; hTERT: human telomerase reverse transcriptase; BDNF: brain-derived neurotrophic factor; TrkB: tropomyosin receptor kinase B; PCNA: proliferating cell nuclear antigen; Wnt5a: Wingless-Type MMTV Integration Site Family, Member 5A; CENP-B: centromere protein B; FOXP3: forkhead box P3; BIM: Bcl2-interacting mediator of cell death; FOXO3a: forkhead box O3a; DDR: DNA damage repair; MDC1: mediator of DNA damage checkpoint protein 1; IFNγ: interferon gamma; CHK1: checkpoint kinase 1; CHK2: checkpoint kinase 2; APC/C: anaphase-promoting complex/cyclosome; TIGIT: T-cell receptor with immunoglobulin and ITIM domains; RANBP1: Ran-specific binding protein 1.
